# The relationship between gait and automated recordings of individual broiler activity levels

**DOI:** 10.1016/j.psj.2021.101300

**Published:** 2021-05-29

**Authors:** Malou van der Sluis, Esther D. Ellen, Britt de Klerk, T. Bas Rodenburg, Yvette de Haas

**Affiliations:** ⁎ Animal Breeding and Genomics, Wageningen University & Research, 6700 AH Wageningen, the Netherlands; † Animals in Science and Society, Faculty of Veterinary Medicine, Utrecht University, 3508 TD Utrecht, the Netherlands; ‡ Cobb Europe, 5831 GH Boxmeer, the Netherlands; § Adaptation Physiology Group, Wageningen University & Research, 6700 AH Wageningen, the Netherlands

**Keywords:** tracking, broiler, activity, gait, ultra-wideband

## Abstract

Gait, or walking ability, is an often-measured trait in broilers. Individual gait scores are generally determined manually, which can be time-consuming and subjective. Automated methods of scoring gait are available, but are often implemented at the group level. However, there is an interest in automated methods of scoring gait at the individual level. We hypothesized that locomotor activity could serve as a proxy for gait of individual broilers. Locomotor activity of 137 group-housed broilers from four crosses was recorded from approximately 16 to 32 days old, using an ultra-wideband tracking system. These birds were divided over four trials. Individual gait scores were determined at the end of the tracking period, on a scale from 0 to 5, with higher scores representing worse gait. Given the limited number of birds, birds were subsequently categorized as having a good gait (GG; scores 0–2) or a suboptimal gait (SG; scores 3–5). Relationships between activity and gait classification were studied to determine whether individual activity has the potential to serve as a proxy for gait. When comparing GG and SG birds using robust linear regression, SG birds showed a lower 1) activity around the start of tracking (estimate = −1.33 ± 0.56, *P* = 0.019), 2) activity near the end of tracking (estimate = −1.63 ± 0.38, *P* < 0.001), and 3) average activity (estimate = −1.12 ± 0.41, *P* = 0.007). When taking day of tracking, trial, cross and body weight category (heavy versus light at approximately 2 wk old) into account, a tendency was still observed for SG birds having lower activity levels within lightweight birds, but not within heavyweight birds. This study provides indications for activity differences between gait classifications. However, given that there was considerable overlap in activity levels between the gait classifications, future research implementing additional activity-related variables is required to allow a more complete distinction between birds with different gait classifications.

## INTRODUCTION

Broiler chickens are often kept in large groups, of several thousands of birds (de Jong et al., 2015). With these large numbers of animals, it can be very complex to observe individual behavior, health, and welfare states. Therefore, there is an increasing interest in easy-to-measure traits that are related to health or welfare states, or to specific behaviors of individual broilers.

An often-measured trait in broilers is their walking ability, or gait, in order to examine leg weakness. Leg weakness is a general term to describe multiple pathological states resulting in impaired walking ability in broilers (Butterworth, 1999). The gait of birds is often classified according to a scoring system developed by Kestin et al. (1992), consisting of 6 categories, ranging from a score of zero that represents a normal gait with no detectable abnormalities to a score of five that represents birds that are incapable of sustained walking on their feet. Side effects of genetic selection, growth rate, body conformation, exercise, stocking density, and other factors have been suggested to be involved in causing leg weakness (reviewed in Bradshaw et al., 2002). Leg weakness has had a considerable prevalence in the conventional broiler industry. In a UK survey by Knowles et al. (2008) it was reported that 27.6% of the birds represented in the survey had a gait score of 3 (i.e., obvious gait defect which affects the ability to move about (Kestin et al., 1992)) or higher at an average age of 40 d, although there was considerable variation between flocks. Leg weakness may negatively affect the birds’ welfare, as there are indications that leg weakness might be painful for the affected birds (Danbury et al., 2000) and, in severe cases, birds may have difficulties competing with others for resources and may be limited in performing specific behaviors (Kestin et al., 1992), such as dustbathing or preening while standing (Vestergaard and Sanotra, 1999; Weeks et al., 2000). Furthermore, lameness can have economic consequences for farmers. For example, in some studies, associations between gait score and footpad dermatitis have been observed in broilers, for example, with high odds of footpad dermatitis becoming more severe as the gait score increases, that is, with a worse gait (Opengart et al., 2018). In the Netherlands, if a threshold of a percentage of birds showing footpad dermatitis is crossed, farmers have to temporarily reduce their stocking densities (Afsprakenkader Implementatie Vleeskuikenrichtlijn, 2009), hereby affecting the farm's economics. It has been suggested that increased locomotor activity can contribute to a lower prevalence of leg weakness (Reiter and Bessei, 2009). Furthermore, different leg health traits have been shown to be heritable (Kapell et al., 2012). For example, tibial dyschondroplasia has been estimated to have a heritability of 0.10 to 0.27 (Kapell et al., 2012). Therefore, information on gait at the level of the individual can be of great value for breeding programs.

Currently, gait scores of individual birds are often determined manually and require an experienced scorer to observe individual birds and grade their walking ability, for example according to the earlier-mentioned 6-scale scoring system from Kestin et al. (1992). However, this manual scoring can be time-consuming and subjective. Therefore, automated ways of estimating, or even predicting, gait scores are desired. Several studies have tested automated ways of scoring gait or expected correlated traits, for example using image technology (e.g., Aydin et al., 2010; Dawkins et al., 2012; Aydin, 2017; Silvera et al., 2017; Nääs et al., 2018; Van Hertem et al., 2018) or inertial measurement units (**IMUs**; e.g., in turkeys; Bouwman et al., 2020). However, the main focus appears to have been on measurements at a group-level. For example, Aydin et al. (2010) implemented an automatic image monitoring system to study activity levels of small groups of birds, clustered based on their manually determined gait score, and observed a relationship between the activity level and the manually determined gait score. They observed that broilers with gait scores 4 and 5 showed the lowest activity levels, although they note that more experiments are needed to assess the repeatability of these findings. Van Hertem et al. (2018) implemented a camera-based automatic animal behavior monitoring tool, to assess, among other things, bird activity levels of flocks, and observed a negative correlation between gait score and flock activity. On the other hand, some automated measurements of individual locomotion have been performed, for example using IMUs (Bouwman et al., 2020). However, although steps could be detected in turkeys with this approach, the relationship with gait score was not studied (Bouwman et al., 2020). Another approach was implemented by Aydin (2017), who manually placed single birds in a test setup with a 3D vision camera system to record the number of lying events and the latency to lie down. Although this has potential to make gait scoring more objective, it was only tested on single birds and in the current setup likely remains a time-consuming and labor-intensive method, as it still requires handling of individual birds for each observation. Therefore, there is a need for a proxy trait that can be used as an indicator for gait score that can be recorded on multiple birds while they are housed in their normal environment. The relationship between gait and the level of locomotor activity of broilers that was reported in some studies (e.g., Aydin et al., 2010; Van Hertem et al., 2018) indicates that the level of locomotor activity at group- or flock-level is correlated with gait and may even have potential as a proxy for gait scores. However, to study the relationship between gait and activity of broilers at the individual level in more detail, individual recordings of gait score and activity are required.

Previous work has shown that the measurement of activity, recorded as distances moved, in broilers can be automated at the individual level (van der Sluis et al., 2019). By tracking activity of individual birds automatically, one can potentially obtain insight into the relationship between activity and gait score of individual birds while they are in a more normal, group-housed situation. If a strong relationship between activity and gait score at the individual level would be observed, activity could potentially be used as a proxy for gait, thereby making scoring of individual birds’ gait more time-efficient and objective. Furthermore, information on activity levels might at the same time be informative for other reasons. For example, activity levels could serve as an indicator of illness, as ill animals often spend more time resting (Gregory, 1998). This renders the collection of activity data at the individual level a potentially fruitful investment.

In this research, data on activity levels, recorded as distances moved, of individual broilers were collected using an ultra-wideband (**UWB**) tracking system and were studied to determine the relationship between individual locomotor activity and gait. Different aspects of individual activity were studied in relation to gait: 1) the activity level at different time points, 2) the overall average activity level, and 3) the slope of activity over time. Furthermore, it was studied whether gait and activity over time were related while accounting for other potentially influential factors, including for example genetic background and body weight of the birds.

## MATERIALS AND METHODS

### Ethical Statement

Data were collected under control of Cobb Europe. Cobb Europe complies with the Dutch law on animal well-being. This study is not considered to be an animal experiment under the Law on Animal Experiments, as confirmed by the local Animal Welfare Body (June 20, 2018, Lelystad, the Netherlands).

### Location and Housing

All data were collected on a broiler farm in the Netherlands. The broilers were group-housed, with feed and water provided ad libitum and wood shavings as bedding. No perches or other additional enrichments were provided. Commercial lighting and temperature schedules were used, and all birds were vaccinated according to common practice (Cobb, 2018).

### Ultra-Wideband Tracking System

A Ubisense UWB system with Series 7000 sensors and compact tags (Ubisense Limited, Cambridge, UK) was used, in combination with TrackLab software (version 1.4, Noldus Information Technology, Wageningen, the Netherlands), to collect data on activity of broilers. The system is described in more detail in van der Sluis et al. (2019). All broilers were fitted with battery-powered UWB tags on their backs, with a size of approximately 3.8 by 3.9 cm and a weight of approximately 25 g, using elastic bands around their wing base. This system recorded the locations of the birds over time, with a frequency of one sample per bird approximately every 6.9 s, and the resulting calculated distances moved of the broilers were used as a measure of individual activity.

### Activity Data Collection

Four consecutive UWB tracking trials (T1–T4) were performed, that is using four production rounds, and activity data were collected on a total of 150 commercial male broiler chickens from 4 different crosses. Not all crosses were present in each trial, as each trial included birds from only 2 crosses, and not all crosses were equally represented in the study (Table 1). At approximately 2-wk-old, the focal birds were selected from a larger group, based on their body weight. This was done to obtain approximately equal samples of lightweight and heavyweight birds within the respective cross and trial. The birds were tracked in a pen with a size of approximately 6 m^2^ in T1, T2, and T4, and in a pen with a size of approximately 8 m^2^ in T3. In all trials, the pen was divided into 2 equal-sized compartments, each housing a single cross. Additional birds from the same line without UWB tags were added before the tracking started in T3 and T4 to increase the housing density to approximately 12 birds/m^2^, compared to a density of approximately 6 birds/m^2^ in T1 and T2. UWB recordings were made from 00:00 to 23:30 each day and the data from 16 to 32 days old (n = 17 d) were used in this study. For T4 there were no data available before 18 d old (n = 15 days of data included for this trial) and in T3 there was a technical issue resulting in no data for 26 and 27 days old (n = 15 days of data included for this trial). Due to too much missing data (see van der Sluis et al. (2019) for details on the data filtering), death of birds and mistakes in sexing, a total sample size of 137 birds was available for analysis. Table 1 shows the weights of the birds in the different weight categories and trials. For these 137 birds, the average distance moved in meters per hour was calculated per day and animal, and was used as the measure of locomotor activity.

**Table 1:** Overview of the weights of the birds in the respective weight categories for the different trials.

T	SW day	EW day	Weight category	Included crosses	SW (g)	EW (g)	Average increase per day (g)
T1	13	34	L (n = 16)	C1 (n = 7); C2 (n = 9)	420 (SD 21)	2,435 (SD 165)	95 (SD 7)
			H (n = 16)	C1 (n = 8); C2 (n = 8)	520 (SD 14)	2,635 (SD 233)	100 (SD 11)
T2	14	33	L (n = 18)	C2 (n = 9); C3 (n = 9)	485 (SD 29)	2,450 (SD 141)	105 (SD 7)
			H (n = 17)	C2 (n = 9); C3 (n = 8)	595 (SD 24)	2,680 (SD 181)	110 (SD 10)
T3	14	35	L (n = 15)	C3 (n = 8); C4 (n = 7)	480 (SD 45)	2,500 (SD 205)	95 (SD 8)
			H (n = 20)	C3 (n = 10); C4 (n = 10)	630 (SD 25)	2,715 (SD 307)	100 (SD 15)
T4	13	35	L (n = 17)	C3 (n = 8); C4 (n = 9)	340 (SD 68)	2,155 (SD 313)	85 (SD 12)
			H (n = 18)	C3 (n = 9); C4 (n = 9)	460 (SD 22)	2,520 (SD 132)	95 (SD 6)

Abbreviations: C1, cross 1; C2, cross 2; C3, cross 3; C4, cross 4; EW, end weight; H, heavyweight; L, lightweight; SW, start weight; T, trial.

Weights of individual birds were determined with 5-gram precision and reported averages are rounded to five-grams. SDs are rounded to whole numbers.

### Gait Scoring

For the gait scores, the data on gait that are routinely collected on this farm were used. Individual gait was determined at 33, 34 or 35 days old, depending on the trial. The gait was determined at 34 days old in T1, at 33 days old in T2, and at 35 days old in T3 and T4. The birds were observed while walking and given a gait score by an experienced human observer. For the different trials, this was not always the same observer, as two observers scored gait during this study. However, scoring within a trial was performed by a single observer. No data on inter-observer reliability was available, but both observers were trained in the same manner, that is, by scoring gait together with an experienced observer until sufficient experience and confidence were developed to start scoring individually. The gait scoring was performed in the pen, but was combined with individual weighing of the birds. Therefore, all birds were handled immediately before gait was scored. Upon placing the birds back in the pen after weighing, their gait was assessed. It must be noted that, as the birds were handled immediately before their gait was scored, stress from the handling may have impacted their gait. However, given that all birds were handled, this potential influence on gait is assumed to be similar for all birds. For the gait scoring, the scoring system shown in Table 2 was used, which is the commonly used system at the farm where the study was conducted. Although this scoring system is not exactly the same as the commonly implemented scoring system from Kestin et al. (1992), the overall idea is similar and for comparing purposes the gait score categories from both scoring systems are assumed to represent similar gaits. The distribution of gait scores is shown in Table 3, where it is also indicated into which weight category the birds were categorized. Given the small sample sizes for some of the gait score categories, a further classification into a ‘good gait’ (**GG**) vs. a ‘suboptimal gait’ (**SG**) was made that was used in the subsequent analyses. The gait score categories 0 to 2 were classified as GG, whereas 3 and higher were classified as SG. This cut-off value was based on the general assumption that with gait score 3 and higher the welfare of the birds is potentially impaired (Kestin et al., 1992). As can be seen from Table 3, this resulted in 79 GG birds and 58 SG birds.

**Table 2:** Gait scoring system used to determine gait scores in this study.

Score	Description	Criteria
0	Walks very well	
1	Walks good / supple	• Controlled • Stands straight on legs
2	Walks relatively well	• Oriented
3	Walks mediocre	• More out of balance • Sits down quickly • Can translocate well but sits down quickly
4	Walks poorly	• Walks with bent legs and waddles • Walks with spread legs • Legs outwards • Wings often hang down
5	Barely walks	• Can only move by also using wings

**Table 3:** Gait score distribution, shown for the two weight categories (see Table 1).

Gait score / classification	Lightweight	Heavyweight	Total
0	0	0	0
1	9	3	12
2	37	30	67
3	11	24	35
4	8	8	16
5	1	6	7
Good gait (gait scores 0-2)	46	33	79
Suboptimal gait (gait scores 3-5)	20	38	58

### Statistics

For all statistics, R version 4.0.2 was used (R Core Team, 2020). The hourly average activity data were not normally distributed and untransformed data were used for the analyses. The slope of individual activity was calculated by means of linear regression, using the hourly average activity per day over the trial per individual. Linear regression models with sum-to-zero contrasts were implemented to study the relationship between gait classification as GG or SG and the following activity measures:

1) Activity at 18 to 20 days old, representing early activity with all trials having data available; average activity over the three days per animal and only including individuals with all three days available (i.e., no days with too many missing samples for an animal, threshold was set at 90% of samples present within each tracking session, see van der Sluis et al. (2019) for details on data filtering), n = 131.

2) Activity at 30 to 32 days old, representing late activity; average activity over the 3 d per animal and only including individuals with all 3 d available, n = 134.

3) Overall average activity level; only including individuals with all days of the respective trial available, n = 120.

4) The slope of activity over time; all animals included regardless of some missing data, n = 137.

Here, each of the activity measures was separately modeled as a linear function of the gait classification only. This was done to gain insight into whether gait classification alone can be linked to activity levels, regardless of differences in genetic background of the birds, their body weight, or the trial in which the birds were recorded. Given that there appeared to be some outliers in the data, robust linear regression models from the robustbase package (Maechler et al., 2020) were used, which are less sensitive to outliers than common linear regression models. To study how gait classification was related to activity levels while accounting for other potentially influential factors, a linear mixed-effects model with sum-to-zero contrasts was implemented, using the lme4 (Bates et al., 2015) and lmerTest (Kuznetsova et al., 2017) packages. For this analysis, a total of 2,160 observations for 137 animals were used. The fixed effects tested were day of tracking, trial, cross, gait classification, start weight category and weight change. The distribution of crosses and start weight categories across trials is indicated in Table 1. Correlated random intercepts and slopes for individual animals were included in the model as random effects. To test the fixed effects, a backward stepwise approach without interactions was used that included all these effects. The resulting terms that were left were all included in 2-way interactions, except for the interaction between cross and trial, as not all crosses were present in multiple trials. Backward selection was then again performed, and both significant effects (*P* < 0.05) and effects showing a tendency (*P* < 0.1) were kept in the model. The resulting final model was

$$Y_{\text{ijklmn}}\;=\mu \;+\beta {\left(\text{DT}\right)}_{i}+C_{j}+T_{k}+SW_{1}+\text{GS}C_{m}+{\left(\beta \left(\text{DT}\right)\;x\;C\right)}_{\text{ij}}+{\left(\beta \left(\text{DT}\right)\;x\;T\right)}_{\text{ik}}+{\left(\beta \left(\text{DT}\right)\;x\;\text{SW}\right)}_{\text{il}}+{\left(\text{SW}\;x\;\text{GSC}\right)}_{\text{lm}}+\left(1+\beta {\left(\text{DT}\right)}_{i}│ID_{n}\right)+e_{\text{ijklmn}}$$

where Y is the average distance moved per hour, µ is the overall mean, β(DT)_i_ is the i^th^ day of tracking (i = 1 to 17), C_j_ is the j^th^ cross (j = 1 to 4), T_k_ is the k^th^ trial (k = 1 to 4), SW_l_ is the l^th^ start weight category (l = light or heavy), GSC_m_ is the classification of gait (m = GG or SG), (β(DT) × C)_ij_ is the interaction between day of tracking and cross, (β(DT) × T)_ik_ is the interaction between day of tracking and trial, (β(DT) × SW)_il_ is the interaction between day of tracking and start weight category, (SW × GSC)_lm_ is the interaction between start weight category and the classification of gait, (1 + β(DT)_i_|ID_n_) is the random effect of the n^th^ animal's intercept and correlated slope, and e_ijklmn_ is the residual term. Given that the 2 crosses within a trial were housed in 2 separate compartments in the tracking pen, there was a possible influence of side of the pen. However, including side of the pen as a fixed effect did not lead to different conclusions regarding the relationship between activity and gait and side of the pen was therefore not included as a fixed effect. No obvious deviations from normality or homoscedasticity were observed upon visual inspection of the residuals of the model. Reported *P*-values for the model estimates were obtained using the lmerTest package (Kuznetsova et al., 2017). The MuMIn package (Barton, 2020) was used to determine the R^2^ values for the model. The ggplot2 (Wickham, 2016) and sjPlot (Lüdecke, 2020) packages were used to make the visualizations. The level of statistical significance was set at 0.05 and results that are reported in the text are rounded to two decimals.

## RESULTS

### Relationship Between Gait Classification and Activity

**Figure 1:**
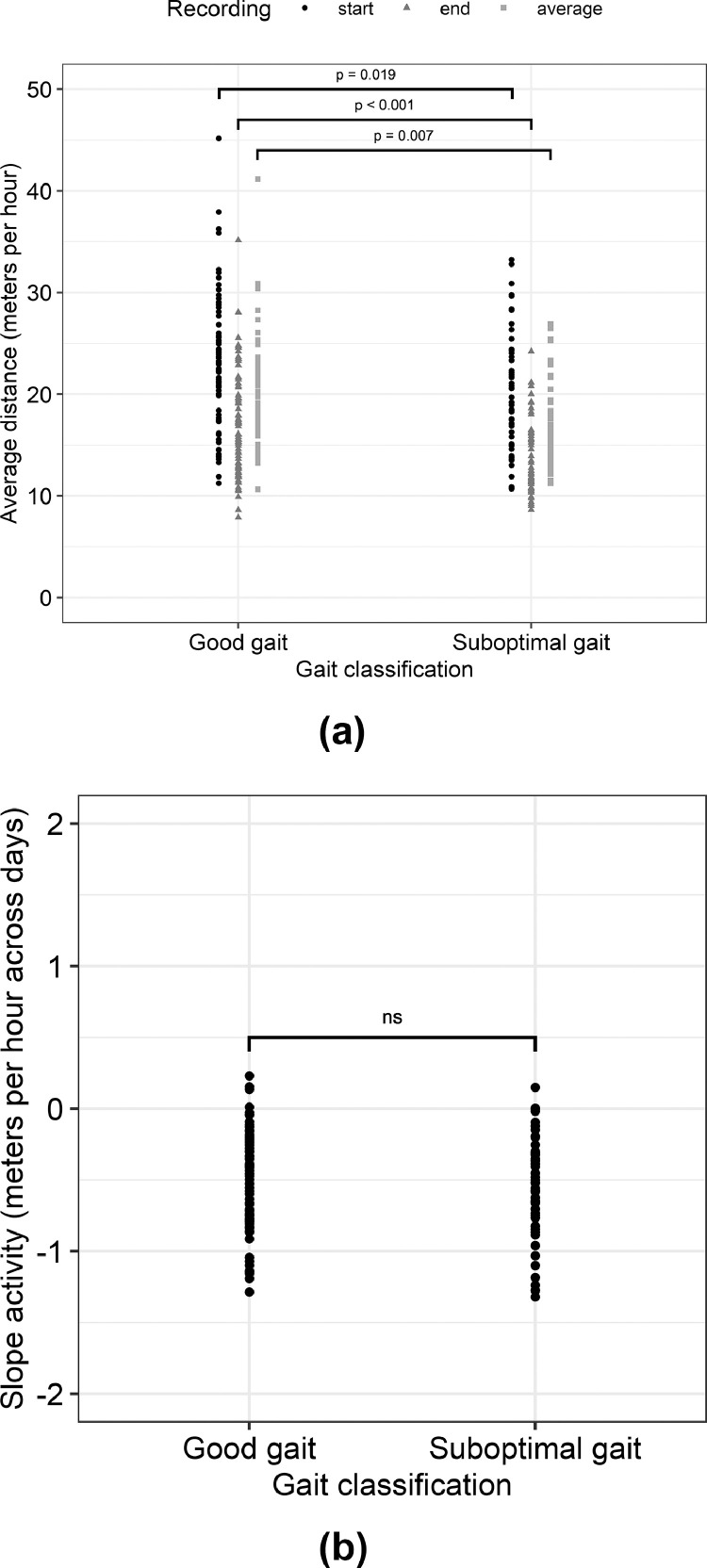
Average distance moved in meters per hour or slope of activity over time for individuals from the different gait classifications. Dots, triangles and squares represent individual data points. (A) start activity as measured at 18 to 20 days old, end activity as measured at 30 to 32 days old, and average activity over the trial; (B) slope of activity over time.

The start activity, as measured at 18 to 20 days old, differed between GG and SG birds, with a higher activity level for GG birds (estimate = 1.33 ± 0.56, *P* = 0.019; Table 4; Figure 1A). This means that on average, GG birds moved 1.33 meters per hour more than the overall average distance recorded at 18 to 20 days old in the study, and thus 2.66 m more than SG birds. The end activity, as measured at 30 to 32 days old, also differed between GG and SG birds, again with a higher activity for GG birds (estimate = 1.63 ± 0.38, *P* < 0.001; Table 4; Figure 1A). The average activity was also higher for GG birds (estimate = 1.12 ± 0.41, *P* = 0.007; Table 4; Figure 1A). No relationship between slope of activity and gait classification was observed (Table 4; Figure 1B).

**Table 4:** Results of the robust linear regression models for the relationship between gait classification and 1) start activity, 2) end activity, 3) average activity, and 4) slope of activity.

Coefficients	Estimate	SE	t-value	Pr(>|t|)
Start activity (Adjusted R2 = 0.037)				
Intercept	21.094	0.576	36.650	<0.001
Gait classification: GG	1.331	0.562	2.369	0.019
End activity (Adjusted R2 = 0.118)				
Intercept	14.888	0.414	35.949	<0.001
Gait classification: GG	1.626	0.378	4.302	<0.001
Average activity (Adjusted R2 = 0.056)				
Intercept	18.000	0.437	41.157	<0.001
Gait classification: GG	1.121	0.412	2.722	0.007
Slope of activity (Adjusted R2 = −0.007)				
Intercept	−0.528	0.032	−16.398	<0.001
Gait classification: GG	0.010	0.032	0.305	0.761

Abbreviation: GG, good gait.

### Relationship Between Gait Classification and Activity in the Presence of Other Influential Factors

**Figure 2:**
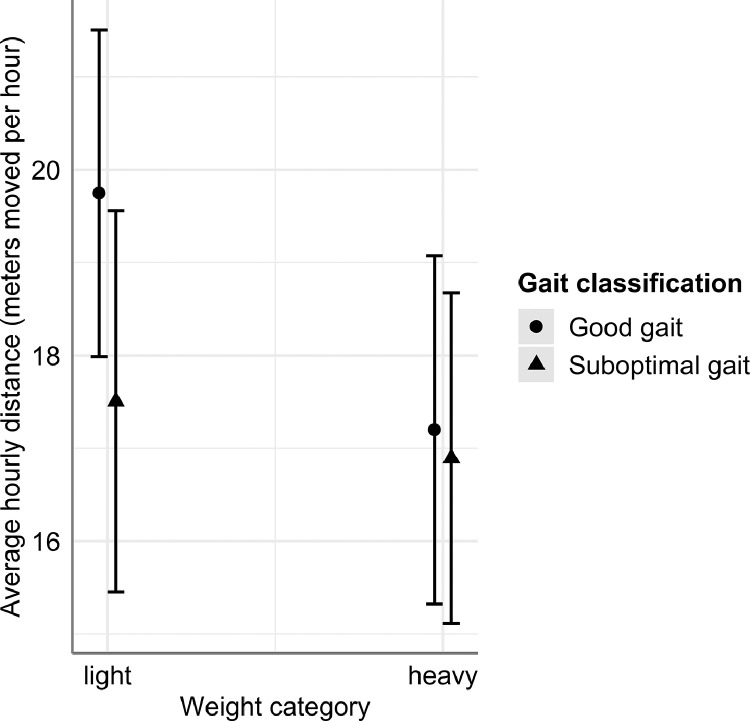
Linear mixed-effects model estimated average hourly distance for good gait and suboptimal gait birds, in interaction with weight category. Bars represent 95% CIs.

To study the effect of GG versus SG on activity levels while taking other possibly influential factors into account, a linear mixed-effects model was implemented (Table 5). This model explained 57.80% of the variance when only fixed effects were included and explained 85.58% of the variance when random effects were included as well. The model showed a tendency for an interaction between gait classification and weight category (Table 5). Within lightweight birds, SG birds appeared to have a lower level of activity than GG birds (Figure 2). This difference between SG and GG birds was not observed within the heavyweight category (Figure 2). Furthermore, a decrease in activity over time was observed, as well as an effect of trial. The degree of the decrease in activity over time differed between trials, crosses, and weight categories.

**Table 5:** Results of the linear mixed-effects model for the predicted average activity (meters moved per hour).

Linear mixed-effects model					
Fixed effects					
Factor	F-value	Pr(>F)	Estimate	SE	Pr(>|t|)
Intercept			24.085	0.495	<2e-16
Day	337.834	<2.2e-16	−0.532	0.029	<2e-16
Trial	27.012	9.66e-14			
- Trial 1			−3.736	1.343	0.006
- Trial 2			−6.218	0.779	6.30e-13
- Trial 3			6.680	0.956	1.21e-10
Cross	2.059	0.109			
- Cross A			3.256	1.465	0.028
- Cross B			0.090	0.913	0.922
- Cross C			−0.907	0.914	0.323
Weight category	11.329	9.86e-04			
- Light			1.353	0.402	9.86e-04
Gait	5.025	0.027			
- Good gait			0.637	0.284	0.027
Day × Trial	19.091	2.18e-10			
- Day × Trial 1			−0.179	0.080	0.026
- Day × Trial 2			0.187	0.046	9.56e-05
- Day × Trial 3			−0.200	0.057	5.46e-04
Day × Cross	3.127	0.028			
- Day × Cross A			0.083	0.087	0.342
- Day × Cross B			0.060	0.054	0.274
- Day × Cross C			0.029	0.054	0.588
Day × Weight category	6.800	0.010			
- Light			−0.061	0.024	0.010
Weight Category × Gait	3.047	0.083			
- Light × Good gait			0.484	0.277	0.083

Random effects					
Factor	Variance	SD	Correlation		
ID intercept	19.245	4.387			
ID by Day	0.059	0.244	−0.75		
Residual	5.707	2.389

## DISCUSSION

In this research, it was studied whether individual levels of activity were related to gait. To this end, the relationships between the individuals’ gait classification and different measures of activity levels were analyzed. Indications for relationships between gait classification and different measures of activity were observed, but gait explained little of the variation in these activity measurements, as R^2^ ranged from 0.04 to 0.12. When taking other possibly influential factors, like day, trial, cross and weight category, into account, a larger part of the variance in activity was explained and a tendency for an interaction between gait classification and weight category was observed. In this interaction, a difference in level of activity was observed between GG and SG in lightweight birds, but not in heavyweight birds.

### Relationship Between Gait Classification and Activity

In this research, a difference between GG and SG birds was observed for several activity measurements. The relationships between gait classification and start activity (18–20 days old), end activity (30–32 days old) and average activity, respectively, indicated that birds with a suboptimal gait showed a lower locomotor activity compared to birds with a good gait. This decrease in activity levels for SG birds, that is, birds with gait score 3 or higher, matches reports in literature in which lower activity levels for birds with higher gait scores were reported (e.g., Weeks et al., 2000; Van Hertem et al., 2018). This often-reported negative relationship between activity level and gait score can have different underlying causes that are difficult to separate from each other. First, it could be the case that the gait itself resulted in the birds limiting their locomotor activity. Several studies have indicated that gait problems might be painful for birds (e.g., McGeown et al., 1999; Danbury et al., 2000) and therefore lame birds might reduce their locomotor activity. On the other hand, it has been suggested that increased locomotor activity may contribute to preventing the development of gait problems (e.g,. Reiter and Bessei, 1998; Reiter and Bessei, 2009). For example, Reiter and Bessei (2009) compared 2 distances between feeders and drinkers, that is, 2 and 12 meters, in broilers. It was observed that the groups of birds with the larger distance had fewer cases of leg weakness and that the locomotor activity in this treatment was higher. Vasdal et al. (2019) performed a pilot study on broiler activity in enriched environments, including peat, bales of lucerne hay, and elevated platforms, and control environments. They observed that the birds in an enriched environment showed higher levels of several activities, for example, ground scratching and ground pecking while standing, and a tendency for a lower gait score than birds in a control environment. Kaukonen et al. (2017) also studied environmental enrichment, as a way to increase activity and thereby improve gait in broilers. They implemented perches and elevated platforms and observed, among other things, lower mean gait scores for the birds in the platform-equipped houses. It was hypothesized that the platform access increased walking to reach the platforms and enabled more versatile movement, which could have positively impacted gait (Kaukonen et al., 2017). Adding elevated platforms or perches, or potentially other types of environmental enrichment, seems a practical approach to improve activity and gait. However, it must be noted that several other studies did not observe a positive effect of perches or platforms on gait (e.g., Bailie and O'Connell, 2015, Baxter et al., 2020). Altogether, birds with higher activity levels might be less prone to gait problems. Moreover, increased activity early in the growing period has been suggested to reduce leg disorders (reviewed in Bradshaw et al., 2002). Unfortunately, in the current study it was not possible to study activity early in life, due to the weight of the tags being a limitation for smaller birds. For future work it would be interesting to look into activity in the first few days of life as well, to gain more insight into the causal relationship between activity level and gait score.

No association between the slope of activity over time and gait classification was observed, suggesting that the difference in activity level between the gait classifications remained relatively constant over time, that is, from 16 to 32 days old. In other words, based on the current data, the activity does not appear to decrease faster over time for SG birds compared to GG birds. It is important to note, however, that the slope values were approached using linear regression, which may have masked some of the nuances in the patterns over time. In a study by Weeks et al. (2000), birds with gaits ranging from gait score 0 to 3 were observed on 6 d between 39 and 49 days old. Exactly which 6 d these were, was not specified further. Although not discussed in detail in their study, when comparing gait score 0 and 1 birds to gait score 2 and 3 birds, it appeared that on the first observation day, the absolute difference in percentage of time spent allocated to walking was smaller compared to d 2 to 5 of observation. However, on d 6 of observation, the absolute difference again appeared to be relatively small. During these 6 recording days, the gait score 2 and 3 birds initially showed a steep decline in the percentage of time that was allocated to walking, but seemed to stabilize over the remainder of the observation period. The gait score 0 and 1 birds showed a more constant decline over these 6 recording days. This suggests that there might be a difference in the activity pattern over time, at least in the period ranging from 39 to 49 days old, which was outside the range of our study period. More research is required to clarify this relationship, preferably over the full life span of broilers and with gait recordings at different time points.

### Relationship Between Gait Classification and Activity in the Presence of Other Influential Factors

In the abovementioned discussion of the relationship between gait classification and activity, other possibly influential factors were not accounted for. Research has indicated that there are relationships between activity and age of the birds (Weeks et al., 2000), weight of the birds (Tickle et al., 2018) and possibly genetics of the birds (Bizeray et al., 2000), respectively. Therefore, in the analysis implementing a linear mixed-effects model, other factors besides gait classification were taken into account. These included time (i.e., age), trial, cross and weight category effects, as well as the interactions between them. Only the main findings related to gait will be discussed here. Results for the other factors have been reported earlier (van der Sluis et al., 2019). Overall, taking the other factors into account still resulted in a tendency for an effect of gait classification being observed, in interaction with weight category. A difference in activity between GG and SG birds appeared to not be present in heavyweight birds, only in lightweight birds (Figure 2). Earlier studies have indicated that birds with higher body weights often walk shorter distances compared to lighter birds, for example in an operant runway test (Bokkers et al., 2007). Also voluntarily, that is, when not necessarily walking for a reward, lightweight birds have been observed to walk longer distances. This was studied for example using weight load reduction, where the weight load on birds’ legs was reduced by partially lifting the birds’ weight using a suspension device (Rutten et al., 2002). A possible explanation for this finding is that as body weight increases, the energetic cost of standing becomes larger than for sitting (Tickle et al., 2018). If heavy birds already limit their activity to the minimally required level to obtain sufficient water and feed, it could be that a suboptimal gait does not decrease the activity level further. Lightweight birds, however, might show activity levels that are higher than required solely for obtaining water and feed. If lightweight birds show a suboptimal gait, this may reduce their activity levels to the level required for solely obtaining water and feed, resulting in an overall decrease in activity. The effect of gait on feeder visits was studied by Weeks et al. (2000). They compared gait score 0 to gait score 3 birds, and observed that gait score 3 broilers visited the feeder less often per day, but increased the visit duration accordingly, resulting in an overall time spent feeding that was similar to that of gait score 0 birds. However, by reducing the number of feeder visits, the distance walked would decrease as well, which could explain the finding in the current study that lightweight birds with a suboptimal gait showed lower distances moved compared to lightweight birds with a good gait.

### Gait Score and Consequences for Welfare

In this research, gait scores of birds were assigned using a 6-scale scoring system. However, given the relatively small sample size, the different gait score categories were later on combined into GG (gait scores 0 to 2) and SG (gait scores 3 to 5) classes for analysis. In this classification of GG versus SG, the cut-off point was positioned between gait scores 2 and 3. This was based on the general assumption that the welfare of birds is potentially impaired at gait score 3 and higher (Kestin et al., 1992). However, it is debatable whether this indeed is a very clear cut-off point. Skinner-Noble and Teeter (2009) compared gait score 2 and gait score 3 birds, and observed among other things that gait score 3 birds stood less and rested more, compared to gait score 2 birds. However, they also studied for example heterophil:lymphocyte ratios as a measure of long-term physiological stress and observed no difference between the 2 groups. Overall, they conclude that there are no indications in their study that the 2 gait score groups differ in their welfare (Skinner-Noble and Teeter, 2009). These findings make it difficult to state where a potential cut-off value may truly lie in terms of welfare. Therefore, if additional research indicates a different cut-off value, it would be advisable for future research to study the relationship between activity levels and the classification GG versus SG based on this new cut-off value.

Furthermore, the different gait scores that comprise each gait classification may differ from each other. For example, gait score 0 is generally described as “[..] walked normally with no detectable abnormality; it was dexterous and agile. [..]”, whereas gait score 2 is generally described as “[..] had a definite and identifiable defect in its gait but the lesion did not hinder it from moving or competing for resources [..]” (Kestin et al., 1992). These 2 gait scores are both classified as GG in this study, but the birds’ behavior and well-being may differ as a consequence of their gait. In our research, it was not possible to study differences between the six gait score categories, due to the limited sample size, but future research with sufficient data on animals from all gait score categories could look into whether it is possible to distinguish each of the 6 gait scores individually, based on activity recordings. This would allow us to assess individual birds’ gait and well-being at a more detailed level.

### Predicting Individual Broiler Gait Using Activity Levels

In this research, we studied the relationship between individual activity and gait classification. Insight into this relationship could, for example, aid in assessing gait of individual birds based on their individual level of activity, which can be recorded in an automated manner. One example of a benefit of this approach for assessing gait is that the possibly confounding effect of stress induced by handling birds, to assess their gait, could be removed. Individual data on broilers’ gait could be informative for many purposes, including for broiler management and for research into the development of gait problems. Furthermore, it has been suggested that some gait problems can be alleviated by selective breeding (reviewed in Bradshaw et al., 2002), which requires data on individual broilers’ gait. It has been reported that out of 3 major broiler breeding companies, at least one implements walking ability, that is, gait, as a trait subject to genetic selection and all select for leg strength (Hiemstra and ten Napel, 2013). A fast way of obtaining gait scores would therefore be beneficial. Moreover, given that it is not feasible for breeding companies to have a single-observer score for all birds, automated gait scoring using activity levels could aid in making gait scoring more objective. However, this study shows that it is difficult to predict the gait score of individual broilers based solely on the here-present activity information, as individual broilers within a gait classification were observed to show quite different activity levels. Furthermore, the observed activity levels within one gait classification showed quite a large overlap with that of the other gait classification, making it difficult to distinguish between gait classifications, and in these models the proportion of the variance in activity that was explained by gait classification was very small. When taking other influential factors into account, a tendency for an interaction between gait classification and weight category was observed. This interaction suggests that activity recordings have the potential to aid in predicting gait of individual birds, when taking other influences on activity levels into account, but that this is only feasible for lightweight birds, as heavyweight birds might already have relatively low activity levels. Overall, it remains difficult to distinguish individual birds’ gait based on distances moved during the period from 16 to 32 days old. Future research could focus on a longer period of time, preferably throughout the entire production period with manual gait recordings periodically implemented, to further study the development of gait problems and the relationship with (early life) activity. Furthermore, additional variables could be studied that are potentially related to gait problems, including, for example, feeder visits (based on findings in Weeks et al., 2000), acceleration and speed of movement (Kestin et al., 1992) and use of the available area. With these additions, automated scoring of individual gait may be feasible, but this remains to be investigated.

In the current setup, the birds were housed in a small pen compared to common broiler housing systems. This potentially resulted in relatively low recorded distances, as activity levels can, for example, be influenced by the distance between feed and water (Reiter and Bessei, 2009), which is likely to be larger in common broiler housing systems. However, in the current study, the focus was on relative activity levels and the differences in activity between GG and SG birds. Therefore, the exact distances moved were not directly of interest. However, Baxter and O'Connell (2020) implemented an UWB system for broiler tracking under commercial conditions and concluded that this was an accurate method for tracking indoor locations of broilers and that, even though absolute distances were generally overestimated, the system can be used to study differences between groups. This suggests that the approach implemented in the current study also has potential for recording activity in larger areas.

## CONCLUSIONS

In this research, it was studied whether individual levels of activity were related to gait of broilers. Indications for relationships between gait classification and different measures of activity were observed, with lower activity levels for birds with a suboptimal gait, but gait explained little of the variation in activity. When taking other possibly influential factors, including day, trial, cross. and weight category into account, a larger part of the variation in activity was explained and a tendency for an interaction between gait classification and weight category was observed. In this interaction, a difference in level of activity was observed between gait classifications in lightweight birds, but not in heavyweight birds. It has to be further investigated if this is a consequence of higher body weight already limiting activity levels. Overall, the differences in activity levels of birds with different gait classifications were not very clear and therefore it remains difficult to distinguish gait classifications based on distances moved during the period from 16 to 32 days old. It is recommended for future studies to look into the relationship between gait and multiple activity-related variables in more detail, throughout the life of broilers, to assess whether automated measures of activity have potential to serve as a proxy for gait at the individual level.

## References

[bib0044] Afsprakenkader Implementatie Vleeskuikenrichtlijn, 2009. Accessed 29 September 2020. https://edepot.wur.nl/12400.

[bib0001] Aydin A. (2017). Using 3D vision camera system to automatically assess the level of inactivity in broiler chickens. Comput. Electron. Agr..

[bib0002] Aydin A., Cangar O., Ozcan S.Eren, Bahr C., Berckmans D. (2010). Application of a fully automatic analysis tool to assess the activity of broiler chickens with different gait scores. Comput. Electron. Agr..

[bib0003] Bailie C.L., O'Connell N.E. (2015). The influence of providing perches and string on activity levels, fearfulness and leg health in commercial broiler chickens. Animal.

[bib0004] Barton, K. 2020. MuMIn: Multi-model inference. R package version 1.43.17. Accessed Aug. 2020. https://CRAN.R-project.org/package=MuMIn.

[bib0005] Bates D., Mächler M., Bolker B.M., Walker S.C. (2015). Fitting linear mixed-effects models using lme4. J. Stat. Softw..

[bib0006] Baxter M., O'Connell N.E. (2020). Testing ultra-wideband technology as a method of tracking fast-growing broilers under commercial conditions. Appl. Anim. Behav. Sci..

[bib0007] Baxter M., Richmond A., Lavery U., O'Connell N.E. (2020). Investigating optimal levels of platform perch provision for windowed broiler housing. Appl. Anim. Behav. Sci..

[bib0008] Bizeray D., Leterrier C., Constantin P., Picard M., Faure J.M. (2000). Early locomotor behaviour in genetic stocks of chickens with different growth rates. Appl. Anim. Behav. Sci..

[bib0009] Bokkers E.A.M., Zimmerman P.H., Rodenburg T.B., Koene P. (2007). Walking behaviour of heavy and light broilers in an operant runway test with varying durations of feed deprivation and feed access. Appl. Anim. Behav. Sci..

[bib0010] Bouwman A., Savchuk A., Abbaspourghomi A., Visser B. (2020). Automated step detection in inertial measurement unit data from turkeys. Front. Genet..

[bib0011] Bradshaw R.H., Kirkden R.D., Broom D.M. (2002). A review of the aetiology and pathology of leg weakness in broilers in relation to welfare. Avian Poult. Biol. Rev..

[bib0012] Butterworth A. (1999). Infectious components of broiler lameness: a review. World's Poultr. Sci. J..

[bib0013] Cobb (2018). Broiler management guide. https://cobbstorage.blob.core.windows.net/guides/5fc96620-0aba-11e9-9c88-c51e407c53ab.

[bib0014] Danbury T.C., Weeks C.A., Chambers J.P., Waterman-Pearson A.E., Kestin S.C. (2000). Self-selection of the analgesic drug carprofen by lame broiler chickens. Vet. Rec..

[bib0015] Dawkins M.S., Cain R., Roberts S.J. (2012). Optical flow, flock behaviour and chicken welfare. Anim. Behav..

[bib0016] De Jong, I. C., H. Gunnink, and V. A. Hindle. 2015. Implementation of the Welfare Quality® broiler assessment protocol – final report. Overview of outcome-based measurement of broiler welfare and a general discussion on the Welfare Quality® broiler assessment protocol. Wageningen, Wageningen UR (University & Research centre) Livestock Research, Livestock Research report 833.

[bib0017] Gregory N.G. (1998). Physiological mechanisms causing sickness behaviour and suffering in diseased animals. Anim. Welf..

[bib0018] Hiemstra, S. J., and J. ten Napel. 2013. Study of the impact of genetic selection on the welfare of chickens bred and kept for meat production. Final report of a project commissioned by the European Commission (DG SANCO 2011/12254).

[bib0019] Kapell D.N.R.G., Hill W.G., Neeteson A.-M., McAdam J., Koerhuis A.N.M., Avendaño S. (2012). Twenty-five years of selection for improved leg health in purebred broiler lines and underlying genetic parameters. Poult. Sci..

[bib0020] Kaukonen E., Norring M., Valros A. (2017). Perches and elevated platforms in commercial broiler farms: use and effect on walking ability, incidence of tibial dyschondroplasia and bone mineral content. Animal.

[bib0021] Kestin S.C., Knowles T.G., Tinch A.E., Gregory N.G. (1992). Prevalence of leg weakness in broiler chickens and its relationship with genotype. Vet. Rec..

[bib0022] Knowles T.G., Kestin S.C., Haslam S.M., Brown S.N., Green L.E., Butterworth A., Pope S.J., Pfeiffer D., Nicol C.J. (2008). Leg disorders in broiler chickens: prevalence, risk factors and prevention. PLoS One.

[bib0023] Kuznetsova A., Brockhoff P.B., Christensen R.H.B. (2017). lmerTest package: tests in linear mixed effects models. J. Stat. Softw..

[bib0024] Lüdecke, D. 2020. sjPlot: data visualization for statistics in social science. R Package Version 2.8.4. Accessed Aug. 2020. https://CRAN.R-project.org/package=sjPlot.

[bib0025] Maechler, M., P. Rousseeuw, C. Croux, V. Todorov, A. Ruckstuhl, M. Salibian-Barrera, T. Verbeke, M. Koller, E. L. T. Conceicao, and M. Anna di Palma. 2020. Robustbase: basic robust statistics. R Package Version 0.93-6. Accessed Aug. 2020. http://CRAN.R-project.org/package=robustbase.

[bib0026] McGeown D., Danbury T.C., Waterman-Pearson A.E., Kestin S.C. (1999). Effect of carprofen on lameness in broiler chickens. Vet. Rec..

[bib0027] Nääs I.de A., Lozano L.C.M., Abdanan Mehdizadeh S., Garcia R.G., Abe J.M. (2018). Paraconsistent logic used for estimating the gait score of broiler chickens. Biosyst. Eng..

[bib0028] Opengart K., Bilgili S.F., Warren G.L., Baker K.T., Moore J.D., Dougherty S. (2018). Incidence, severity, and relationship of broiler footpad lesions and gait scores of market-age broilers raised under commercial conditions in the southeastern United States. J. Appl. Poult. Res..

[bib0029] R Core Team (2020). R: a language and environment for statistical computing.

[bib0030] Reiter K., Bessei W. (1998). Effect of locomotor activity on bone development and leg disorders in broilers. Arch. Geflügelk..

[bib0031] Reiter K., Bessei W. (2009). Effect of locomotor activity on leg disorder in fattening chicken. Berl. Munch. Tierarztl. Wochenschr..

[bib0032] Rutten M., Leterrier C., Constantin P., Reiter K., Bessei W. (2002). Bone development and activity in chickens in response to reduced weight-load on legs. Anim. Res..

[bib0033] Silvera A.M., Knowles T.G., Butterworth A., Berckmans D., Vranken E., Blokhuis H.J. (2017). Lameness assessment with automatic monitoring of activity in commercial broiler flocks. Poult. Sci..

[bib0034] Skinner-Noble D.O., Teeter R.G. (2009). An examination of anatomic, physiologic, and metabolic factors associated with well-being of broilers differing in field gait score. Poult. Sci..

[bib0035] Tickle P.G., Hutchinson J.R., Codd J.R. (2018). Energy allocation and behaviour in the growing broiler chicken. Sci. Rep..

[bib0037] van der Sluis M., de Klerk B., Ellen E.D., de Haas Y., Hijink T., Rodenburg T.B. (2019). Validation of an ultra-wideband tracking system for recording individual levels of activity in broilers. Animals.

[bib0038] Van Hertem T., Norton T., Berckmans D., Vranken E. (2018). Predicting broiler gait scores from activity monitoring and flock data. Biosyst. Eng..

[bib0039] Vasdal G., Vas J., Newberry R.C., Moe R.O. (2019). Effects of environmental enrichment on activity and lameness in commercial broiler production. J. Appl. Anim. Welf. Sci..

[bib0040] Vestergaard K.S., Sanotra G.S. (1999). Relationships between leg disorders and changes in the behaviour of broiler chickens. Vet. Rec..

[bib0041] Weeks C.A., Danbury T.D., Davies H.C., Hunt P., Kestin S.C. (2000). The behaviour of broiler chickens and its modification by lameness. Appl. Anim. Behav. Sci..

[bib0042] Wickham H. (2016). ggplot2: Elegant Graphics for Data Analysis.

